# Where are we now? A multicountry qualitative study to explore access to pre-antiretroviral care services: a precursor to antiretroviral therapy initiation

**DOI:** 10.1136/sextrans-2016-052970

**Published:** 2017-06-14

**Authors:** Dominic Bukenya, Alison Wringe, Mosa Moshabela, Morten Skovdal, Robert Ssekubugu, Sara Paparini, Jenny Renju, Estelle McLean, Oliver Bonnington, Joyce Wamoyi, Janet Seeley

**Affiliations:** 1 Medical Research Council/ Uganda Virus Research Institute, Uganda Research Unit on AIDS, Entebbe, Uganda; 2 Department of Epidemiology and Population Health, London School of Hygiene & Tropical Medicine, London, UK; 3 Africa Health Research Institute, KwaZulu-Natal, South Africa; 4 University of KwaZulu Natal, KwaZulu Natal, South Africa; 5 University of Copenhagen, Copenhagen, Denmark; 6 Biomedical Research and Training Institute, Harare, Zimbabwe; 7 Rakai Health Sciences Program, Rakai, Uganda; 8 Department of Global Health, London School of Hygiene & Tropical Medicine, London, UK; 9 Malawi Epidemiology and Intervention Research Unit, Karonga, Malawi; 10 National Institute for Medical Research, Mwanza, Tanzania

**Keywords:** Africa, HIV, treatment, Social Science

## Abstract

**Objective:**

To explore barriers and facilitators to accessing postdiagnosis HIV care in five sub-Saharan African countries.

**Methods:**

In-depth interviews were conducted with 77 people living with HIV (PLHIV) in pre-antiretroviral therapy care or not-yet-in care and 46 healthcare workers. Participants were purposely selected from health and demographic surveillance sites in Karonga (Malawi), Manicaland (Zimbabwe), uMkhanyakude (South Africa), Kisesa (Tanzania) and Rakai and Kyamulibwa (Uganda). Thematic content analysis was conducted, guided by the constructs of affordability, availability and acceptability of care.-

**Results:**

*Affordability:* Transport and treatment costs were a barrier to HIV care, although some participants travelled to distant clinics to avoid being seen by people who knew them or for specific services. Broken equipment and drug stock-outs in local clinics could also necessitate travel to other facilities.

*Availability:* Some facilities did not offer full HIV care, or only offered all services intermittently. PLHIV who frequently travelled complained that care was seldom available to them in places they visited.

*Acceptability:* Severe pain or sickness was a key driver for accessing postdiagnosis care, whereas asymptomatic PLHIV often delayed care-seeking. A belief in witchcraft was a deterrent to accessing clinical care following diagnosis. Changing antiretroviral therapy guidelines generated uncertainty among PLHIV about when to start treatment and delayed postdiagnosis care. PLHIV reported that healthcare workers’ knowledge, attitudes and behaviours, and their ability to impart health education, also influenced whether they accessed HIV care.

**Conclusion:**

Despite efforts to decentralise services over the past decade, many barriers to accessing HIV care persist. There is a need to increase sustained access to care for PLHIV not yet on treatment, with initiatives that encompass biomedical aspects of care alongside considerations for individual and collective challenges they faced. A failure to do so may undermine efforts to achieve universal access to antiretroviral therapy.

**Key messages**

- Factors influencing access to preantiretroviral therapy and care can be grouped into those relating to affordability, availability and accessibility.
- Most barriers to accessing HIV care in sub-Saharan Africa have persisted over the course of the past decade, despite initiatives to increase service provision.
- Interventions are needed to promote access to postdiagnosis HIV care that encompass biomedical aspects of care alongside considerations for individual and collective challenges faced by people living with HIV (PLHIV).

## Introduction

Writing in 2007, Anita Hardon and colleagues^1^ observed that people living with HIV (PLHIV) in their study areas in Botswana, Tanzania and Uganda faced a number of barriers gaining access to HIV care and antiretroviral therapy (ART). They listed such constraints as ‘transport costs, user fees, long waiting times, hunger, stigma, side-effects, lack of appropriate counselling and workloads’ (p. 660). In the introduction to their seminal paper, Hardon *et al* remind us of the July 2005 goal set by the G8 meeting at the Gleneagles Summit to provide ‘as close as possible’ universal access to ART to all those in need by 2010.^2^ In the decade since the publication of Hardon’s paper, barriers to accessing HIV care and ART persist, despite notable strides being made towards achieving this universal access goal, with improved availability of testing and treatment in many African countries. In particular, many PLHIV who are asymptomatic are not entering care after receiving an HIV diagnosis.^3^ As discussed in the editorial of this volume,^4^ changes in treatment guidelines pose a challenge to the goal of achieving wider access to HIV care. As more people become eligible for ART, health systems struggle to keep up with demand.^3 5–7^ Yet, while shortages of staff or drugs may affect access for those who reach health services, additional obstacles continue to undermine that very access itself. In a systematic review focusing on retention in care between HIV testing and treatment access, Rosen and Fox^8^ note that there are ‘substantial’ losses from pre-ART care partly because people may not come back to clinic to collect ART, despite being eligible. With this in mind, in this paper, we investigate the extent to which the barriers to linkage to care identified by Hardon *et al* 1 are still present. An understanding of access barriers is crucial given global health priorities to expand to universal access to ART.

Drawing on data from five countries, and using a ‘framework of access’ developed by McIntyre *et al* 9 to evaluate general healthcare access and applied by Cleary *et al* 10 in the context of access to ART, we group and expand on the barriers to access identified by Hardon *et al*. McIntyre’s framework provides a structure through which to assess the affordability, availability and acceptability of services. As Cleary *et al* explain (p.142), ‘affordability’ is the fit between the costs of utilising a service and the patient’s ability to pay associated costs; ‘availability’ is the fit between a patient’s needs and type, place and time of services provided; and ‘acceptability’ is the fit between healthcare provider and patient attitudes and expectations of each other.

## Methods

### Study setting

Data for this paper were drawn from the Bottlenecks Study^4^ covering six health and demographic surveillance sites across five countries in Eastern and Southern Africa within the ALPHA network (http://alpha.lshtm.ac.uk/). More information can be found in the online methods supplement found at http://dx.doi.org/10.1136/sextrans-2017-053172.^11^ The study sites were: Karonga (Malawi), Manicaland (Zimbabwe), uMkhanyakude (South Africa), Kisesa (Tanzania), and Rakai and Kyamulibwa (Uganda). In the five countries, national policies are in place to provide free HIV testing through a variety of models and those diagnosed HIV-positive are referred for care to a government-run clinic, except for Kyamulibwa where referral is to either a government or non- governmental clinic. All but one (Karonga) of the surveillance sites have the capacity to follow individuals from diagnosis through care initiation and monitor subsequent HIV care engagement.

### Sampling frame and participants

PLHIV were purposively sampled to include those who had enrolled in pre-ART care (accessing co-trimoxazole) and those tested but not yet enrolled into care. Only in the Kyamulibwa site were PLHIV in pre-ART care randomly sampled due to large numbers. In the rest of the sites, PLHIV and in all sites, healthcare workers (HCWs) (eg, nurses and medical assistants) were purposively recruited into the study.

### Data collection, management and analysis

Data were generated through face-to-face in-depth interviews (IDIs), conducted by trained social science interviewers. The interviews were held in the participants’ homes/places of work/other venues of their choice and lasted between 60 and 90 min. IDIs with PLHIV covered the individual circumstances influencing HIV testing uptake, enrolment and retention in pre-ART HIV care. IDIs with HCWs focused on their experiences of offering HIV care and circumstances affecting people’s enrolment into care. All IDIs were audio-recorded. Detailed interview summaries with illustrative quotations were developed at the Kyamulibwa site and analysed manually. In other sites, audio-recorded interviews were transcribed and translated into English before importing into Nvivo V.8/10. All data were coded and analysed thematically, using deductive and inductive logic by teams at each site. The site lead researchers produced detailed analytical summaries from emerging themes and subthemes from the analysis. The lead author reviewed the summaries and developed an analytical coding scheme based on elements of the access framework described above. There were regular discussions with in-country study coordinators to ensure emerging findings agreed with data interpretation.

### Ethical considerations

The study received ethics approval in each of the participating countries and from the ethics committee at London School Hygiene and Tropical Medicine. Prior to data collection participants were provided with an information sheet on what the study was about and opportunities to ask questions. All participants gave written informed consent.

## Results

A total of 123 people participated in the study (77 PLHIV and 46 HCWs, see table 1).

**Table 1:** Number of participants per study site and participant category

Category	Karonga, Malawi	Kisesa, Tanzania	Kyamulibwa, Uganda	Rakai, Uganda	uMkhanyakude, South Africa	Manicaland, Zimbabwe	Total
Diagnosed no ART	9	13	8	15	16	16	77
No contact with care/ineligible	5	4	4	5	10	6	39
Eligible/pre-ART	4	9	4	10	6	10	38
Healthcare workers	5	7	5	6	19	4	46
Total	14	20	13	21	35	20	123

ART, antiretroviral therapy.

### Affordability

Both patients and HCWs reported transport cost as a major barrier to access free HIV services in all study sites, except Zimbabwe, compounded in some places (Malawi, Tanzania and Uganda) by the distance that had to be travelled for services where free treatment was available.

*The government health facilities are located far. You need to spend about 20,000/= ($5-$6) on a return journey. […] you cannot get affordable services around here.* (Female, PLHIV on Pre-ART, Uganda)

In Zimbabwe, it was reported that HIV service decentralisation had removed this access barrier. In other places, outreach clinics had made services more accessible for some by offering pre-ART testing, health education and co-trimoxazole/Bactrim drug refills. Participants in Tanzania and Uganda recalled a time when transport had usefully been provided for those in serious need, and home visits for follow-up made, but funding cuts had since affected that provision.

Yet at all sites, HIV stigma influenced the distance travelled for testing and postdiagnosis care. Some people preferred to travel to distant places to avoid being seen by people who knew them. Additional costs were incurred if these more distant facilities were private clinics. In all the sites, participants mentioned that securing funds for both the trip and the private service could delay access to care.

Others had to travel for specific services. Pregnant women in Kyamulibwa, Uganda, for example, had to access Option B+ at more distant public facilities because the local clinic did not provide antenatal care. In Malawi and Zimbabwe, CD4 testing pre-ART sometimes had to be sought from distant facilities because of faulty testing machines at the local clinic.

*They said you should take your medication on time and constantly get CD4 count. However, the machine for the CD4 count is not working at this clinic.[…] I came here last month and heard that the machine was down and you should go [elsewhere].* (Female, PLHIV-diagnosed, not in care, Zimbabwe)

In addition to broken equipment, drug stock-outs in local clinics could also necessitate travel to more distant facilities, which may make the service unaffordable and therefore unavailable. Next, we explore availability further.

### Availability

In all but one site (Rakai, Uganda), PLHIV reported that access to pre-ART care services was delayed or unsuccessful because some health facilities did not offer all the HIV care components, or offered them intermittently. Some facilities in Malawi, Tanzania and Zimbabwe lacked CD4 testing machines (or, as noted above, the machine was broken).

*We don’t test for the CD4 here but after referring them to xxx we just follow him to find out if he went, so if someone went to xxx we ask him to give us what was recorded.* (HCW, Malawi)

Interestingly, some delays in access to care were attributed by respondents not to the lack of services, but to the lack of follow-up from HCWs to get PLHIV into care. However, in most of the sites, some provision for follow-up was offered. PLHIV were actively followed up in some cases through home visits and/or SMS messaging and outreach services. However, in most sites, follow-up had reduced in recent years because of funding cuts.

Availability of services was also affected by the mobility of PLHIV, particularly in Malawi, Tanzania and both Kyamulibwa and Rakai in Uganda where many people travelled for work. PLHIV explained that clinics in the surveillance sites targeted specific catchment areas and when moving to areas outside the surveillance site, they became ineligible to be served by those clinics, with potential interruptions in care. PLHIV explained that these interruptions happened because they could not get refills of their pre-ART drug (co-trimoxazole/Bactrim). They also frequently complained that if they tried to go to a clinic in the area they were visiting, they would have to start the process of testing and assessment afresh to get the service they wanted. The inaccessibility of postdiagnosis services to visiting non-residents of clinic catchment areas was also reported by HCWs, although sometimes HCWs did help visiting PLHIV.

*I had gone to nurse my late mother with my drugs and they got finished while I was still in xxx. I decided to go in xxx referral hospital with my treatment cards [from local clinic] and showed them to the nurses. I told them that I was getting the drugs from xxx and that they were now remaining only three tablets. They gave me drugs for one month and when I came back here, I went to [the clinic] and reported it then I was given drugs for one month and given another appointment.* (Female, PLHIV pre-ART, Uganda)

The availability issues above highlight leakages in the HIV care continuum at every stage: linking to care, starting ART and sustaining ART access. Gaining and maintaining access to ART also depend on the acceptability of the services available.

### Acceptability

In Malawi, Uganda and Zimbabwe, many PLHIV and HCWs reported that having severe pain/sickness was one of the main motivators for seeking postdiagnosis care. Those who were asymptomatic saw little reason to seek care.

*I was not sick during that time; my body was just healthy. Even though I tested positive my body was just healthy and I didn’t also suffer from any sickness.* (Female, PLHIV- on Pre- ART, Malawi)

PLHIV and HCWs in Malawi, South Africa and Uganda reported that a belief in witchcraft was a major deterrent to accessing postdiagnosis care because health conditions linked to witchcraft required traditional healing. Therefore, the treatment offered at clinics was not acceptable and indeed, for some people, using a drug from a clinic to treat a traditional disease could impede recovery.

Beliefs about witchcraft could be reinforced by family and friends.

*I had these swellings in the neck and he (brother) would tell me that you probably ate some things that were stolen. And yet I had never stolen anything in my life. So I was really confused, some people would say that I was possessed by a local god and it used to just confuse me all the more. That day I was going to a different traditional doctor when they told me that I have the virus and that is what was making me sick.* (Male, PLHIV pre-ART, Uganda)

Some HCWs in South Africa and Uganda reported recognising the benefits of the updated ART guidelines, but to some extent found the changes confusing and difficult to justify to patients. This generated uncertainty among their patients about when to start treatment, which contributed to delays in HIV postdiagnosis care. HCWs advocated for more resources to provide accurate information on what is available in terms of HIV care and for whom. HCWs further noted that some PLHIV needed more time than others to come to terms with their HIV status prior to accepting HIV treatment and may thus wish to postpone treatment initiation.

*Counselling must be deep so that it will last for a long time in a patient’s mind, and that way they can’t drop out of pre-ART services or treatment. […] The kind of patients that are not ready are those who hold on to things like’ X infected me’ or ‘Y told me to nurse her sick child’! […] The ready patients are those that accept their status and forget about ‘who stole whose man’, and so forth and take the treatment.* (HCW, South Africa)

While PLHIV and HCWs reported well-managed and coordinated HIV care services at the surveillance site clinics, with good records on those accessing services (including pre-HIV and post-HIV counselling), some considered ‘too much monitoring’ an obstacle to sustained attendance. For example, there were concerns that insisting on short times between refills of drugs affected the acceptability of care. This was echoed by some HCWs.

*The major challenge among new cases is that we give them short periods to return. […] So you have seen a client who’s new, he even still fears coming to the clinic because it’s for positive people but they have to come back for bleeding […] ‘frequent visits’ […] can challenge them because most people come from far.* (HCW, Uganda)

However, it was also the case that short intervals between drug refills were sometimes due to HCWs managing a shortage in drug supplies. HCWs also noted that some people appreciated short gaps between clinic visits if they needed encouragement to take their treatment.

Across the sites, PLHIV reported that the quality of HCWs’ knowledge and their ability to impart health education influenced the acceptability of care. Matters were not helped by counsellors’ explanations that participants could not understand (sero-discordance, and different guidelines for pregnant women and general population were mentioned), which made them doubt the competence of the clinic staff overall. Dissatisfaction with health education or counselling was also reported in Malawi, Tanzania, and Kyamulibwa, in Uganda.

HCWs’ behaviour also impacted on acceptability in Malawi, South Africa, Tanzania and Uganda. Some HCWs were reported to be rude, which frightened patients away. Furthermore, breaches in confidentiality, with HCWs disclosing who had tested HIV positive and who had been initiated onto treatment, put PLHIV off attending services.

*Some doctors or nurses should go for further training in order to learn how to handle patients especially those with AIDS. […] patients need much care and not to be harassed or shouted at.* (Female, PLHIV not in care, Uganda)

However, the acceptability of care was enhanced whenever support was available. PLHIV in all the study sites reported that they appreciated receiving psychological support to encourage testing and engagement in care and/or economic support.

*After they counselled me, I was encouraged to go to the xxx and start medicine. I have now realised that the counselling that I was given was beneficial to me* (Male, PLHIV pre-ART, Malawi)

Material, as well as psychological, support often came from relatives and friends but also from HCWs who occasionally helped patients they felt were in need.

Some PLHIV, in all sites, associated being unmarried and living alone with a lack of support and encouragement, which they reported as a main cause of their delayed access to pre-ART care. However, interestingly, some participants in Tanzania said that being single had motivated them to seek healthcare, because they knew they would have no one to help them if they were to become very ill. And, in many sites, married PLHIV mentioned that unsupportive partners delayed their access to care.

*I only got support from the counsellor but there was no support from the partner.* (Female, PLHIV-on ART, Uganda)

Where peer groups of PLHIV existed, these were not always considered supportive. In Malawi, newly diagnosed PLHIV commented that it was hard to join existing groups, and some PLHIV in South Africa said that they had lost enthusiasm for support groups over time.

The acceptability of treatment affected access to care among those diagnosed with HIV. A change in service provision (eg, a less supportive health worker or change in clinic services) could render ART access less acceptable, even if it were both affordable and available.

## Discussion

**Figure 1:**
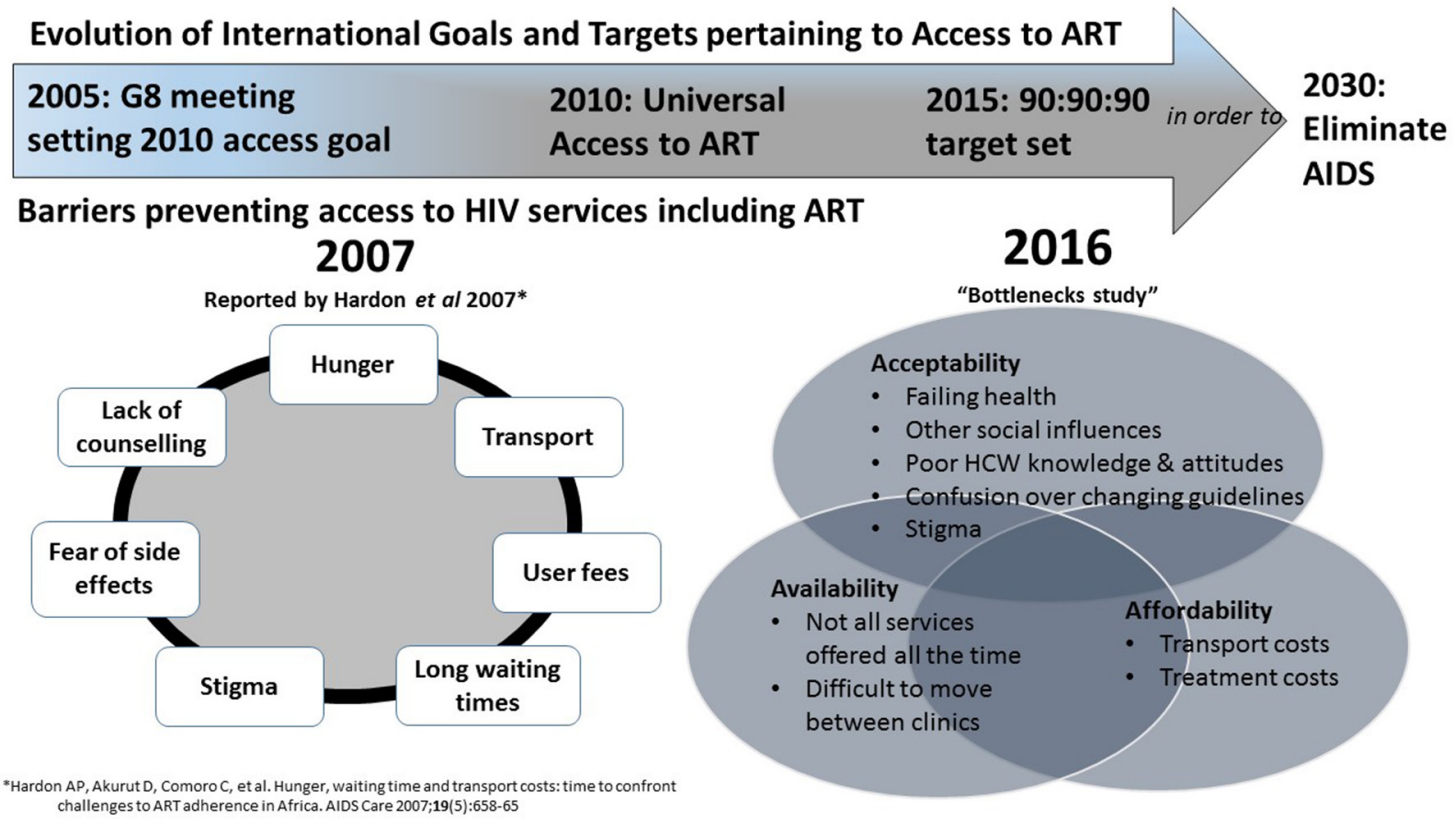
Evolution of International Goals and ART Access Targets and barriers to ART access.

The reasons given by PLHIV in our study for not accessing postdiagnosis care echo the earlier findings of Hardon *et al* 1 (see figure 1). Despite increased decentralisation of treatment, distance, equipment breakdown and transport costs still matter, with revisions to guidelines increasing the number of people eligible for treatment. Stigma and fears over the personal and familial consequences of starting on treatment also remain important, as demonstrated in other settings.^12–15^

Moreover, our findings suggest that the interaction between these barriers may be more important than the singular role played by each factor. The affordability and availability of services interact in complex ways and are affected by the acceptability of care across contexts. If someone fears being seen at their local clinic, for example, that care is unacceptable and unavailable, perhaps resulting in an unaffordable cost to them seeking care elsewhere.

As the findings of the ANRS 12249 TasP Trial have shown,^16^ innovative approaches of linking people to care are needed, if universal ‘test and treat’ is to succeed. Much can be learnt from demographic surveillance studies that will be relevant to healthcare systems in general. Healthcare systems in similar resource-poor settings should strengthen linkages with social services, and HCWs should be trained to recognise and address social and economic concerns faced by PLHIV. Furthermore, demonstrating patient-centredness in the services provided to PLHIV would improve quality of care in the health services. Initiatives that aim to integrate HIV care with other services^17^ may provide a way forward in tackling some of these barriers to entry and retention in postdiagnosis care.

## Conclusion

There is a need to increase access to and retention in HIV care for PLHIV, with initiatives that focus on biomedical aspects of HIV care, such as monitoring the CD4 count or viral load, and that consider individual and collective challenges faced by PLHIV. As calls for ‘test and start’ increase, growing numbers of asymptomatic PLHIV will require tailored support and care, alongside an assured drug supply.
